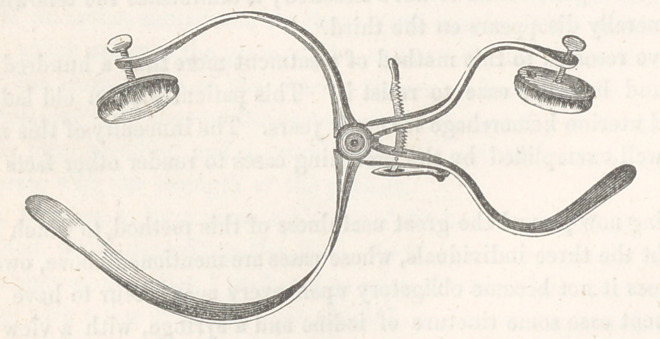# Account of a New Tourniquet

**Published:** 1857-01

**Authors:** S. D. Gross

**Affiliations:** Professor of Surgery in the Jefferson Medical College of Philadelphia


ART. VI.—*An Account of a New Tourniquet.* By S. D. GROSS, M.D., Professor of Surgery in the Jefferson Medical College of Philadelphia.

A great variety of tourniquets are before the profession. Indeed, one would suppose that there was no end to the invention and modification of such instruments. A large volume would hardly suffice for their delineation and description. Every one is acquainted with the tourniquet of J. L. Petit, one of the earliest, most simple, and most perfect of the old instruments of this kind, and which a modern surgeon eulogized by saying that it was insusceptible of improvement, and that no other tourniquet could ever take its place. This prophecy has proved to be untrue. The instrument of Petit, notwithstanding its many excellencies, is not always easy of adjustment, or of convenient management, and, on more occasions than one, it has suddenly snapped asunder in the midst of an operation, much to the detriment of the patient and the horror of the surgeon. It may be said that such an occurrence is due more to the materials of which the instrument is composed, than to the manner of its construction; this may be so, and yet the fact does not invalidate the force of the objection. No good instrument should be liable to such a contingency. The last few years have supplied us with the tourniquets of Signoroni and Skey, two valuable instruments, now much in vogue, especially in Europe.

The tourniquet which I have recently invented, and of which the accompanying cut is an accurate representation, possesses, if I mistake not, decided advantages over any now in use; first, in the facility of its application; secondly, in the amount of pressure which it is capable of exerting; thirdly, in its ready adaptation to limbs of different sizes; fourthly, in the circumstance that it makes pressure only at two points, that is, over the artery and at the point immediately opposite to the artery; and, lastly, the facility with which it may be slackened or removed at any stage of the operation. With a slight modification, the instrument could be readily adapted to the femoral artery as it emerges from beneath Poupart's ligament, or even to the external iliac just above this ligament, in amputation at the hip-joint, and also to the axillary artery, in disarticulation of the shoulder-joint.

By a reference to the cut, drawn by Mr. Daniels, it will be seen that the instrument is composed of two blades, differing in the degree of their curvatures, united by a screw, and regulated by a rachet. Each short blade is provided with a pad, capable of being worked by a screw, and designed to rest upon the artery which it is intended to compress. By this arrangement two tourniquets are produced: a large one for the thigh, and a small one for the arm, or the thigh of a small subject. I will only add, that the instrument was manufactured at my suggestion by Mr. Kolbe, an eminent cutler of this city.

## Figures and Tables

**Figure f1:**